# Metal-Resistance in Bacteria: Why Care?

**DOI:** 10.3390/genes11121470

**Published:** 2020-12-08

**Authors:** Raymond J. Turner, Li-Nan Huang, Carlo Viti, Alessio Mengoni

**Affiliations:** 1 Department of Biological Sciences, Faculty of Science, University of Calgary, Calgary, AB T2N 1N4, Canada; 2 School of Life Sciences, Sun Yat-Sen University, Guangzhou 510275, China; eseshln@mail.sysu.edu.cn; 3 Laboratorio Genexpress, Dipartimento di Scienze e Tecnologie Agrarie, Alimentari, Ambientali e Forestali, Università di Firenze, 50144 Florence, Italy; carlo.viti@unifi.it; 4 Laboratorio di Genetica Microbica, Dipartimento di Biologia, Università di Firenze, 50019 Florence, Italy

Heavy metal resistance is more than the tolerance one has towards a particular music genera. The study of metal resistance mechanisms in bacteria traces back to the 1970s and through to the mid 1990s. During these early days, specific metal or metalloid ion resistance determinants, consisting of single metal(loid) resistance genes (MRGs) to large complex operons, were being identified on large conjugative plasmids and other mobile genetic elements. These determinants were often used to classify these accessory plasmid components of genomes. Thinking back to a conference on this topic with speakers from our ancestors of this field, such as Simon Silver, Ann Summers, Barry Rosen, Diane Taylor, Geoff Gadd, Dietrich Nies, and Max Mergeay, who were presenting their work of cloning, sequencing, and characterizing metal resistance in microbes at this time. This early work performed in the pre-omics’ era made great strides in exploring bacteria response to silver, nickel, cadmium, mercury, copper, arsenite/arsenate, and tellurite. It was not that long before it was realized that metal resistance in bacteria essentially follows a limited number of biochemical processes [1,2,3], e.g., prevention of metals’ uptake; if it gets in; efflux it back out again; sequestration through metal binding proteins or chelating metabolites; oxidation-reduction to change redox state or other chemical modification (either removal or addition of organic constituents) to change the metal’s speciation; sequestration through precipitation to metal crystal form or the production of metal binding proteins or chelating metabolites. These seem like trivial statements to say today, but a remarkable amount of work has been put forward to understand such processes at the genetic, biochemical, and structural biology levels. Yet, even with the power of omics approaches, there are still many metal-microbe interaction puzzles left to solve.

The work exploring specific metal-resistance determinants has been complemented by those researchers exploring the ability of various bacterial species to respire using different metal(loids) as electron donors or acceptors [4,5]. Additionally, the studies on metal resistance over the past 50 years, derived primarily from the clinical environment, are complemented by the work evaluating the microbiology of extreme environments, from deep sea vents to mine drainage/tailings and industrial sites, evaluating bacteria’s role in geochemistry. This work gave us the multi-metal resistant *Cupriavidus metallidurans*, which has become an important model organism in this regard [6]. The genomics of this species strains have provided us amazing insight into how bacteria can survive high metal loads and how bacteria can survive anthropogenically abused environments. It would be impossible to deny the advances in knowledge that the genomic revolution has given our field. Even as early as the late 2000s, it took 3 years of work to sequence a strain to obtain a rough draft of an aluminum resistant polychlorinated biphenyl (PCBs) degrading strain [7]. At the time, getting this information was remarkable, and this sequenced genome sparked new hypothesis and important findings. On reflection, it could take 6 months to sequence an operon in 1990. Now, of course, sequencing and assembling a genome can be done in a week. Multiple strains can be sequenced and their genomes compared for unique single nucleotide polymorphisms (SNPs) and gene operon changes. Bioinformatic mining of genomes allows for an understanding of specific genetic traits related to metals [8]. Beyond sequencing, other omic approaches have evolved and been applied to the field of metal resistance in bacteria, including proteomic (example [9]), metabolomic [10,11], and comparative genomics approaches [12], methods of chemical genomics [13], or the comprehensive approach of resistance metalloproteomics [14]. Combining various omics together to look at the response of the transcriptome, proteome, and metabolome by metals is referred to as metallomics [15].

So why do we care, or why should we care, about metal-resistance in bacteria? The research directions described above still continue in labs around the world, but now more often focus on the advent of biotechnological or bioremediation advances. Over the past decade, we have seen the knowledge of metal resistance in bacteria be used in the eco-friendly production of a wide variety of metal nanomaterials [16]. The appreciation of the normal sensitivity of most bacteria to several metals has led to a resurgence of their use as metal(loid)-based antimicrobials [17,18] as a result of moving into the antimicrobial resistance era and the need for new and novel antimicrobials. As such, we have also seen an exponential use of metal(loid)-based nanoparticles used as antimicrobial agents [19]. Of course, resistance has already started to develop against different metal nanomaterial formulations [20].

Through the journey from the 1970s, we have obtained a good view of the acquired MRGs. It is now reasonably well established that many are found on mobile genetic elements and genomic islands similar to antibiotic resistant genes (ARGs). Using modern day genomics, we can see beyond the specific gene determinants and toward the full system responses of metal challenges to bacteria. We have begun to see various global regulator systems, such as MarR [21], providing regulated tolerance to both antibiotics and metals. Similarly, we see multidrug resistance efflux pumps providing co-resistance to metals, antiseptics, and antibiotics [22]. This also helps us to understand the link between the use of metal ions in agriculture practices and its influence on the world’s antimicrobial resistance challenges [23].

It was through the variety of genomic approaches that we found the genes, metabolic pathways, and key enzymes involved in resistance and tolerance mechanisms in bacteria. Yet knowledge gaps exist in our understanding of bacterial sensitivity to metal challenges. How do naïve bacterial species respond to metal stress? Can we see metal resistance develop in real-time? Our various anthropogenic activities have led to metal resistance bacteria in aquatic and marine environments [24]. This is beginning to allow us to understand how bacteria survive acute metal ion challenges as well as chronically living under constant metal exposed aggression.

We have learned a lot about metal-resistance to date. What does the future hold in this field that genomics tools will feed? As metabolic modeling of microbes improves [25], how will our view and use of metal-resistance in bacteria change? Pontification here gives possibilities of novel metal(loid) respiring species, bioremediation strategies for the many metal polluted sites world-wide, novel metal-based antimicrobial treatments, biocatalysts in green chemistry, understanding of bacterial evolution in relationship to the Earth’s geological history, and modelling natural selection of microbial communities and microbial strains.

The present Special Issue, which includes two reviews [26,27], two featured papers [28,29], and eight original manuscripts [30,31,32,33,34,35,36,37] covers many of the above-mentioned aspects of genomics in bacteria resistance. The review papers discuss the knowledge and perspective of heavy-metal resistance in human pathogens and in the challenge of plant symbiotic microbiome exploitation in phytoremediation of heavy-metal polluted soils. The research papers present novel data on the genetics of resistance in model and pathogenic species and in biotechnologically relevant strains. Witnessing the need to still fully understand the genetics and evolution of heavy-metal resistance novel work on the previously mentioned model bacterium *C. metallidurans* is also presented.
